# *Neostagonosporellasichuanensis* gen. et sp. nov. (Phaeosphaeriaceae, Pleosporales) on *Phyllostachysheteroclada* (Poaceae) from Sichuan Province, China

**DOI:** 10.3897/mycokeys.46.32458

**Published:** 2019-02-18

**Authors:** Chun-Lin Yang, Xiu-Lan Xu, Dhanushka N. Wanasinghe, Rajesh Jeewon, Rungtiwa Phookamsak, Ying-Gao Liu, Li-Juan Liu, Kevin D. Hyde

**Affiliations:** 1 College of Forestry, Sichuan Agricultural University, Wenjiang District, Huiming Road 211, Chengdu 611130, Sichuan, China Sichuan Agricultural University Chengdu China; 2 Forestry Research Institute, Chengdu Academy of Agricultural and Forestry Sciences, Nongke Road 200, Chengdu 611130, Sichuan, China Mae Fah Luang University Chiang Rai Thailand; 3 Center of Excellence in Fungal Research, Mae Fah Luang University, Chiang Rai, 57100, Thailand Forestry Research Institute, Chengdu Academy of Agricultural and Forestry Sciences Chengdu China; 4 Key Laboratory for Plant Diversity and Biogeography of East Asia, Kunming Institute of Botany, Chinese Academy of Science, Kunming 649201, Yunnan, China Kunming Institute of Botany, Chinese Academy of Science Kunming China; 5 Department of Health Sciences, Faculty of Science, University of Mauritius, Reduit, Mauritius University of Mauritius Reduit Mauritius

**Keywords:** 2 new taxa, bambusicolous fungi, phylogeny, stem spot, taxonomy

## Abstract

*Neostagonosporellasichuanensis* sp. nov. was found on *Phyllostachysheteroclada* collected from Sichuan Province in China and is introduced in a new genus *Neostagonosporella* gen. nov. in this paper. Evidence for the placement of the new taxon in the family Phaeosphaeriaceae is supported by morphology and phylogenetic analysis of a combined LSU, SSU, ITS and TEF 1-α DNA sequence dataset. Maximum-likelihood, maximum-parsimony and Bayesian inference phylogenetic analyses support *Neostagonosporella* as a distinct genus within this family. The new genus is compared with related genera of Phaeosphaeriaceae and full descriptions and illustrations are provided. *Neostagonosporella* is characterised by its unique suite of characters, such as multiloculate ascostromata and cylindrical to fusiform, transversely multiseptate, straight or curved ascospores, which are widest at the central cells. Conidiostromata are multiloculate, fusiform to long fusiform or rhomboid, with two types conidia; macroconidia vermiform or subcylindrical to cylindrical, transversely multiseptate, sometimes curved, almost equidistant between septa and microconidia oval, ellipsoidal or long ellipsoidal, aseptate, rounded at both ends. An updated phylogeny of the Phaeosphaeriaceae based on multigene analysis is provided.

## Introduction

The family Phaeosphaeriaceae is a large and important family of Pleosporales, initially introduced by Barr (1979) with *Phaeosphaeriaoryzae* I. Miyake as the type species (Miyake 1909). The taxonomy of members within this family has often been confused with those of the Leptosphaeriaceae (Müller 1950, Holm et al. 1957, Munk 1957, Zhang et al. 2009, Phookamsak et al. 2014) and it is sometimes difficult to distinguish species. Criteria which have previously been used to differentiate species have been based mostly on the morphology of the peridial wall, asexual characteristics and host association (Eriksson 1967, 1981, Lucas and Webster 1967, Leuchtmann 1984, Shoemaker 1984, Barr 1987, Shoemaker and Babcock 1989, Shearer et al. 1990, Khashnobish and Shearer 1996, Câmara et al. 2002) and taxonomic schemes followed are those of Kirk et al. (2008), Zhang et al. (2009), Hyde et al. (2013), Phookamsak et al. (2014a) and Abd-Elsalam et al. (2016). However, this delimitation of taxa in Phaeosphaeriaceae and Leptosphaeriaceae, based solely on the above-mentioned features, is not feasible. Recent studies showed that it is very difficult to discriminate them only by such characters, because numerous new members have been introduced to these two families and these species are not significantly different in these features, but they can be differentiated by phylogenetic analysis (Zhang et al. 2012, Hyde et al. 2013, Ahmed et al. 2014, Ariyawansa et al. 2015a, 2018, Bakhshi et al. 2018). Hence there is a need to use the multigene sequence data analyses to infer relationships.

Barr (1979) originally introduced 15 genera in this family and subsequent researchers have revised this number (Barr 1992, Eriksson and Hawksworth 1993, Kirk et al. 2001, 2008, Lumbsch and Huhndorf 2007, 2010). The taxonomic placement of genera within this family has been changed in recent years based on phylogenetic analyses (Zhang et al. 2012, Hyde et al. 2013, Wijayawardene et al. 2014, Phookamsak et al. 2014a, 2017, Wanasinghe et al. 2018). Taxonomic revision of the genera in Phaeosphaeriaceae resulted in 28 genera based on morphology and phylogenetic evidence (Phookamsak et al. 2014a). Since 2014, many new genera have been introduced based on molecular data (Ariyawansa et al. 2015b, Ertz et al. 2015, Crous et al. 2015a, 2015b, 2017a, Jayasiri et al. 2015, Li et al. 2015, Phukhamsakda et al. 2015, Rossman et al. 2015, Tibpromma et al. 2015, 2017, Abd-Elsalam et al. 2016, Hernández-Restrepo et al. 2016, Hyde et al. 2016, 2017, Tennakoon et al. 2016, Wijayawardene et al. 2016, Ahmed et al. 2017, Huang et al. 2017, Karunarathna et al. 2017, Phookamsak et al. 2017, Bakhshi et al. 2018, Senanayake et al. 2018, Wanasinghe et al. 2018). The placement of some older genera has been reconfirmed with DNA sequence (Phookamsak et al. 2017, Senanayake et al. 2018). However, there are still a few genera lacking molecular data, such as *Bricookea*, *Dothideopsella*, *Eudarluca*, *Phaeostagonospora* and *Tiarospora*. At present, this family includes more than 800 species in 61 genera (25 genera are known only from asexual morphs) (Index Fungorum 2018, Wijayawardene et al. 2017, 2018). Many genera were introduced to accommodate a single or a few species in Phaeosphaeriaceae. Only 14 genera in the Phaeosphaeriaceaecontained 10–50 species, while *Ophiobolus* and *Phaeosphaeria* comprised more than 150 species. However, most species in *Ophiobolus* and *Phaeosphaeria* lack molecular data to confirm their phylogenetic affinities.

We are studying fungi on bamboo which is the main food for panda in Sichuan Province of China (Tang et al. 2007, Wang et al. 2017). The purpose of this paper is to introduce a new genus with one species in Phaeosphaeriaceae recovered from *Phyllostachysheteroclada* Oliv. Combined multigene (LSU, SSU, ITS and TEF 1-α) analyses confirm its phylogenetic position in Phaeosphaeriaceae. A comprehensive comparison with similar genera and detailed descriptions and illustrations are provided.

## Materials and methods

### Sampling and morphological study

The specimens were collected from Ya’an City of Sichuan Province in China, on living to near dead stems and branches of *Phyllostachysheteroclada*. The samples were kept in Ziplock plastic bags and brought to the laboratory. Fresh materials were examined by using stereo and compound microscopes. Vertical free-hand sections were made by using a razor blade and placed on a droplet of sterilised water on a glass slide (Gupta and Tuohy 2013). Lactate cotton blue reagent was used to observe the number of septa. Micro-morphological characters were examined by using a Nikon ECLIPSE N*i* compound microscope fitted to a Cannon 600D digital camera. Fruiting tissues were observed by stereomicroscopy using NVT-GG (Shanghai Advanced Photoelectric Technology Co. Ltd, China) and photographed by VS-800C (Shenzhen Weishen Times Technology Co. Ltd, China). Measurements were taken using Tarosoft® Image Frame Work v.0.9.7.

### Isolation

Single ascospore and conidium isolation was carried out following the method described by Dai et al. (2017). Germinated ascospores and conidia were separately transferred to Potato Dextrose Agar media plates (PDA) and incubated at 25°C and the colonies were observed after 10 days and as outlined by Vijaykrishna et al. (2004) and Liu et al. (2010). Specimens are deposited in Mae Fah Luang University Herbarium (MFLU), Chiang Rai, Thailand and Sichuan Agricultural University Herbarium (SICAU), Chengdu, China. Living cultures are deposited at the Culture Collection at Mae Fah Luang University (MFLUCC) and the Culture Collection at Sichuan Agricultural University (SICAUCC). Facesoffungi and Index Fungorum numbers were registered as in Jayasiri et al. (2015) and Index Fungorum (2018), respectively. New species are established following the recommendations of Jeewon and Hyde (2016).

### DNA extraction, PCR amplification and sequencing

Fungal isolates were grown on PDA for seven days at 25°C and genomic DNA was extracted from fresh mycelia, following the protocols of Plant Genomic DNA Kit (Tiangen, China). If cultures were unavailable, fungal DNA was directly extracted from fruiting tissues according to Yang et al. (2017), Wanasinghe et al. (2018) and Zeng et al. (2018). The primers, LR0R and LR5 (Vilgalys and Hester 1990), NS1 and NS4, ITS5 and ITS4 (White et al. 1990) and EF1-983F and EF1-2218R (Rehner 2001) were used for the amplification of the 28S large subunit rDNA (LSU), 18S small subunit rDNA (SSU), internal transcribed spacers (5.8S, ITS) and translation elongation factor 1-α gene region (TEF 1-α), respectively. The amplification reactions were performed as stated by Phukhamsakda et al. (2015). Amplified PCR fragments were purified and sequenced at TsingKe Biological Technology Co., Ltd. (Chengdu, China). Newly generated sequences of LSU, SSU, ITS and TEF 1-α regions are deposited in GenBank.

### Molecular phylogenetic analysis

Sequence data, mainly from recent publications (Phookamsak et al. 2017, Wanasinghe et al. 2018), were downloaded for analyses (Table 1). Four Massarineae taxa *Cyclothyriellarubronotata* (CBS 121892), *C.rubronotata* (CBS 141486), *Didymosphaeriarubi-ulmifolii* (MFLUCC 14-0024) and *D.variabile* (CBS 120014) were chosen as outgroup taxa based on Tanaka et al. (2015) and Jaklitsch and Voglmayr (2016). DNA alignments were performed by using MAFFT v.7.407 online service (Katoh and Standley 2013) and ambiguous regions were excluded with BioEdit version 7.0.5.3 (Hall 1999). Multigene sequences were concatenated by Mesquite version 3.11 (build 766) (Maddison and Maddison 1997–2016). Multigene phylogenetic analyses of the combined LSU, SSU, ITS and TEF 1-α sequence data were obtained from maximum likelihood (ML), maximum parsimony (MP) and Bayesian inference (BI) analyses. The alignments were converted to NEXUS file (.nxs) by using ClustalX version 1.81 (Thompson et al. 1997) for MP and BI analyses. The symbols “ABCDEFGHIKLMNOPQRSTUVWXYZ” was deleted in PAUP v. 4.0b10 (Swofford 2002) for preparing data matrix of evaluated evolutionary model by MrModeltest v. 2.2 (Nylander 2004). The best nucleotide substitution model was determined by MrModeltest v. 2.2 (Nylander 2004) and the best-fit model for BI is GTR+I+G under the Akaike Information Criterion (AIC).

**Table 1.** Molecular data used in this study and GenBank accession numbers.

Species	Strain/Voucher No.	GenBank Accession No.				Refferences
		LSU	SSU	ITS	TEF 1-α	
*** Acericola italica ***	**MFLUCC 13-0609**	MF167429	MF167430	MF167428	-	Hyde et al. 2017
* Allophaeosphaeria muriformia *	MFLUCC 13-0277	KX910089	KX950400	KX926415	-	Liu et al. 2015
*** Allophaeosphaeria muriformia ***	**MFLUCC 13-0349**	KP765681	KP765682	KP765680	-	Liu et al. 2015
*** Amarenographium ammophilae ***	**MFLUCC 16-0296**	KU848197	KU848198	KU848196	MG520894	Wijayawardene et al. 2016, Phookamsak et al. 2017
*** Amarenomyces dactylidis ***	**MFLUCC 14-0207**	KY775575	-	KY775577	-	Hyde et al. 2017
* Ampelomyces quisqualis *	CBS 131.31	JX681066	-	AF035781	-	Kiss and Nakasone 1998, Verkley et al. 2014
* Ampelomyces quisqualis *	CBS 133.32	JX681067	-	-	-	Verkley et al. 2014
*** Banksiophoma australiensis ***	**CBS 142163**	KY979794	-	KY979739	-	Crous et al. 2017
*** Bhatiellae rosae ***	**MFLUCC 17-0664**	MG828989	MG829101	MG828873	-	Wanasinghe et al. 2018
* Boeremia exigua *	CBS 431.74	EU754183	EU754084	FJ427001	GU349080	Aveskamp et al. 2009, de Gruyter et al. 2009, Schoch et al. 2009
*** Camarosporioides phragmitis ***	**MFLUCC 13-0365**	KX572345	KX572350	KX572340	KX572354	Hyde et al. 2016
*** Chaetosphaeronema achilleae ***	**MFLUCC 16-0476**	KX765266	-	KX765265	-	Hyde et al. 2016
*** Chaetosphaeronema hispidulum ***	**CBS 216.75**	KF251652	EU754045	KF251148	-	de Gruyter et al. 2009, Quaedvlieg et al. 2013
* Cyclothyriella rubronotata *	CBS 121892	KX650541	-	KX650541	KX650516	Jaklitsch and Voglmayr 2016
*** Cyclothyriella rubronotata ***	**CBS 141486**	KX650544	KX650507	KX650544	KX650519	Jaklitsch and Voglmayr 2016
*** Dactylidina shoemakeri ***	**MFLUCC 14-0963**	MG829003	MG829114	MG828887	MG829200	Wanasinghe et al. 2018
*** Dematiopleospora cirsii ***	**MFLUCC 13-0615**	KX274250	-	KX274243	KX284708	Hyde et al. 2016
*** Dematiopleospora fusiformis ***	**MFLU 15-2133**	KY239030	KY239028	KY239029	-	Huang et al. 2018
*** Dematiopleospora mariae ***	**MFLUCC 13-0612**	KJ749653	KJ749652	KJ749654	KJ749655	Wanasinghe et al. 2014
* Didymocyrtis caloplacae *	CBS 129338	JQ238643	-	JQ238641	-	Lawrey et al. 2012
* Didymocyrtis ficuzzae *	CBS 128019	JQ238616	-	KP170647	-	Lawrey et al. 2012, Trakunyingcharoen et al. 2014
*** Didymocyrtis xanthomendozae ***	**CBS 129666**	JQ238634	-	KP170651	-	Lawrey et al. 2012, Trakunyingcharoen et al. 2014
*** Didymosphaeria rubi-ulmifolii ***	**MFLUCC 14-0024**	KJ436585	KJ436587	-	-	Ariyawansa et al. 2014
* Didymosphaeria variabile *	CBS 120014	JX496139	-	JX496026	-	Verkley et al. 2014
*** Dlhawksworthia alliariae ***	**MFLUCC 13-0070**	KX494877	KX494878	KX494876	-	Hyde et al. 2016
*** Dlhawksworthia clematidicola ***	**MFLUCC 14-0910**	MG829011	MG829120	MG828901	MG829202	Wanasinghe et al. 2018
*** Dlhawksworthia lonicera ***	**MFLUCC 14-0955**	MG829012	MG829121	MG828902	MG829203	Wanasinghe et al. 2018
* Dothidotthia aspera *	CPC 12933	EU673276	EU673228	-	-	Phillips et al. 2008
*** Dothidotthia symphoricarpi ***	**CPC 12929**	EU673273	EU673224	-	-	Phillips et al. 2008
* Edenia gomezpompae *	AM04	KM246015	-	KM246160	-	González et al. 2007
*** Edenia gomezpompae ***	**CBS 124106**	FJ839654	-	FJ839619	-	Crous et al. 2009
*Edenia* sp.	UTHSC: DI16-264	LN907407	-	LT796858	LT797098	Valenzuela-Lopez et al. 2017
*Edenia* sp.	UTHSC: DI16-260	LN907403	-	LT796855	LT797095	Valenzuela-Lopez et al. 2017
*** Embarria clematidis ***	**MFLUCC 14-0652**	KT306953	KT306956	KT306949	-	Ariyawansa et al. 2015a
* Embarria clematidis *	MFLUCC 14-0976	MG828987	MG829099	MG828871	MG829194	Wanasinghe et al. 2018
*** Equiseticola fusispora ***	**MFLUCC 14-0522**	KU987669	KU987670	KU987668	MG520895	Abd-Elsalam et al. 2016, Phookamsak et al. 2017
* Foliophoma fallens *	CBS 161.78	GU238074	GU238215	KY929147	-	Aveskamp et al. 2010, Crous and Groenewald 2017
* Foliophoma fallens *	CBS 284.70	GU238078	GU238218	KY929148	-	Aveskamp et al. 2010, Crous and Groenewald 2017
*** Galiicola pseudophaeosphaeria ***	**MFLU 14-0524**	KT326693	-	KT326692	MG520896	Phookamsak et al. 2017
*** Italica achilleae ***	**MFLUCC 14-0959**	MG829013	MG829122	MG828903	MG829204	Wanasinghe et al. 2018
*** Juncaceicola italica ***	**MFLUCC 13-0750**	KX500107	KX500108	KX500110	MG520897	Phookamsak et al. 2017
*** Juncaceicola luzulae ***	**MFLUCC 13-0780**	KX449530	KX449531	KX449529	MG520898	Tennakoon et al. 2016, Phookamsak et al. 2017
*** Leptospora galii ***	**KUMCC 15-0521**	KX599548	KX599549	KX599547	MG520899	Phookamsak et al. 2017
* Leptospora rubella *	CPC 11006	DQ195792	DQ195803	DQ195780	-	Crous et al. 2006
*** Leptospora thailandica ***	**MFLUCC 16-0385**	KX655549	KX655554	KX655559	KX655564	Hyde et al. 2016
* Loratospora aestuarii *	JK 5535B	GU301838	GU296168	-	-	Schoch et al. 2009
*** Melnikia anthoxanthii ***	**MFLUCC 14-1010**	KU848204	KU848205	-	-	Wijayawardene et al. 2016
"***Muriphaeosphaeria" ambrosiae***	**MFLU 15-1971**	KX765264	-	KX765267	-	Hyde et al. 2016
*** Muriphaeosphaeria galatellae ***	**MFLUCC 14-0614**	KT438329	KT438331	KT438333	-	Phukhamsakda et al. 2015
* Muriphaeosphaeria galatellae *	MFLUCC 15-0769	KT438330	KT438332	-	-	Phukhamsakda et al. 2015
*** Neocamarosporium lamiacearum ***	**MFLUCC 17-560**	MF434279	MF434367	MF434191	MF434454	Wanasinghe et al. 2017
*** Neosetophoma clematidis ***	**MFLUCC 13-0734**	KP684153	KP684154	KP744450	-	Liu et al. 2015
* Neosetophoma rosae *	MFLUCC 17-0844	MG829035	MG829141	MG828926	MG829219	Wanasinghe et al. 2018
*** Neosetophoma rosae ***	**MFLU 15-1073**	MG829034	MG829140	MG828925	MG829218	Wanasinghe et al. 2018
*** Neosphaerellopsis thailandica ***	**CPC 21659**	KP170721	-	KP170652	-	Trakunyingcharoen et al. 2014
*** Neostagonospora arrhenatheri ***	**MFLUCC 15-0464**	KX910091	KX950402	KX926417	MG520901	Phookamsak et al. 2017, Thambugala et al. 2017
*** Neostagonospora caricis ***	**CBS 135092**	KF251667	-	KF251163	-	Quaedvlieg et al. 2013
*** Neostagonospora phragmitis ***	**MFLUCC 16-0493**	KX910090	KX950401	KX926416	MG520902	Phookamsak et al. 2017, Thambugala et al. 2017
*** Neostagonosporella sichuanensis ***	**MFLUCC 18-1228**	MH368073	MH368079	MH368088	MK313851	This study
* Neostagonosporella sichuanensis *	MFLUCC 18-1231	MH368074	MH368080	MH368089	-	This study
* Neostagonosporella sichuanensis *	MFLU 18-1223	MH394690	MH394687	MK296469	MK313854	This study
*** Neosulcatispora agaves ***	**CPC 26407**	KT950867	-	KT950853	-	Crous et al. 2015b
*** Nodulosphaeria guttulatum ***	**MFLUCC 15-0069**	KY496726	KY501115	KY496746	KY514394	Tibpromma et al. 2017
*** Nodulosphaeria multiseptata ***	**MFLUCC 15-0078**	KY496728	KY501116	KY496748	KY514396	Tibpromma et al. 2017
*** Nodulosphaeria scabiosae ***	**MFLUCC 14-1111**	KU708846	KU708842	KU708850	KU708854	Mapook et al. 2016
*** Ophiobolopsis italica ***	**MFLUCC 17-1791**	MG520959	MG520977	MG520939	MG520903	Phookamsak et al. 2017
*** Ophiobolus artemisiae ***	**MFLUCC 14-1156**	KT315509	MG520979	KT315508	MG520905	Phookamsak et al. 2017
* Ophiobolus artemisiae *	MFLU 15-1966	MG520960	MG520978	MG520940	MG520904	Phookamsak et al. 2017
*** Ophiobolus disseminans ***	**MFLUCC 17-1787**	MG520961	MG520980	MG520941	MG520906	Phookamsak et al. 2017
*** Ophiobolus italicus ***	**MFLUCC 14-0526**	KY496727	-	KY496747	KY514395	Tibpromma et al. 2017
*** Ophiobolus rossicus ***	**MFLU 17-1639**	MG520964	MG520983	MG520944	MG520909	Phookamsak et al. 2017
* Ophiobolus rudis *	CBS 650.86	GU301812	AF164356	KY090650	GU349012	Liew et al. 2000, Schoch et al. 2009, Ahmed et al. 2016
*** Ophiobolus senecionis ***	**MFLUCC 13-0575**	KT728366	-	KT728365	-	Tibpromma et al. 2015
*** Ophiosimulans tanaceti ***	**MFLUCC 14-0525**	KU738891	KU738892	KU738890	MG520910	Tibpromma et al. 2016b, Phookamsak et al. 2017
* Ophiosphaerella agrostidis *	MFLUCC 11-0152	KM434281	KM434290	KM434271	KM434299	Phookamsak et al. 2014a
* Ophiosphaerella agrostidis *	MFLUCC 12-0007	KM434282	KM434291	KM434272	KM434300	Phookamsak et al. 2014a
*** Ophiosphaerella aquatica ***	**MFLUCC 14-0033**	KX767089	KX767090	KX767088	MG520911	Ariyawansa et al. 2015a, Phookamsak et al. 2017
*** Paraleptosphaeria rubi ***	**MFLUCC 14-0211**	KT454718	KT454733	KT454726	-	Ariyawansa et al. 2015b
*** Paraophiobolus arundinis ***	**MFLUCC 17-1789**	MG520965	MG520984	MG520945	MG520912	Phookamsak et al. 2017
*** Paraophiobolus plantaginis ***	**MFLUCC 17-0245**	KY815010	KY815012	KY797641	MG520913	Hyde et al. 2017, Phookamsak et al. 2017
*** Paraphoma chrysanthemicola ***	**CBS 522.66**	GQ387582	GQ387521	KF251166	-	de Gruyter et al. 2010, Quaedvlieg et al. 2013
*** Paraphoma radicina ***	**CBS 111.79**	KF251676	EU754092	KF251172	-	de Gruyter et al. 2009, Quaedvlieg et al. 2013
*** Parastagonospora dactylidis ***	**MFLUCC 13-0375**	KU058722	-	KU058712	-	Li et al. 2015
*** Parastagonospora italica ***	**MFLUCC 13-0377**	KU058724	MG520985	KU058714	MG520915	Li et al. 2015, Phookamsak et al. 2017
*** Parastagonospora minima ***	**MFLUCC 13-0376**	KU058723	MG520986	KU058713	MG520916	Li et al. 2015, Phookamsak et al. 2017
*** Parastagonospora uniseptata ***	**MFLUCC 13-0387**	KU058725	MG520987	KU058715	MG520917	Li et al. 2015, Phookamsak et al. 2017
*** Parastagonosporella fallopiae ***	CBS 135981	MH460545	-	MH460543	-	Bakhshi et al. 2018
* Parastagonosporella fallopiae *	CCTU 1151.1	MH460546	-	MH460544	-	Bakhshi et al. 2018
*** Phaeopoacea festucae ***	**MFLUCC 17-0056**	KY824767	KY824769	KY824766	-	Thambugala et al. 2017
* Phaeopoacea phragmiticola *	CBS 459.84	KF251691	KY090700	KF251188	-	Quaedvlieg et al. 2013, Ahmed et al. 2016
*** Phaeosphaeria acaciae ***	**MFLUCC 17-0320**	KY768868	KY768870	KY768869	-	Hyde et al. 2017
*** Phaeosphaeria chiangraina ***	**MFLUCC 13-0231**	KM434280	KM434289	KM434270	KM434298	Phookamsak et al. 2014a
* Phaeosphaeria musae *	MFLUCC 11-0151	KM434278	KM434288	KM434268	KM434297	Phookamsak et al. 2014a
*** Phaeosphaeria oryzae ***	**CBS 110110**	KF251689	GQ387530	KF251186	-	de Gruyter et al. 2010, Quaedvlieg et al. 2013
*** Phaeosphaeria thysanolaenicola ***	**MFLUCC 10-0563**	KM434276	KM434286	KM434266	KM434295	Phookamsak et al. 2014a
*** Phaeosphaeriopsis dracaenicola ***	**MFLUCC 11-0157**	KM434283	KM434292	KM434273	KM434301	Phookamsak et al. 2014a
*** Phaeosphaeriopsis glaucopunctata ***	**MFLUCC 13-0265**	KJ522477	KJ522481	KJ522473	MG520918	Thambugala et al. 2014, Phookamsak et al. 2017
*** Phaeosphaeriopsis triseptata ***	**MFLUCC 13-0271**	KJ522479	KJ522484	KJ522475	MG520919	Thambugala et al. 2014, Phookamsak et al. 2017
* Phoma herbarum *	AFTOL-ID 1575	DQ678066	DQ678014	-	DQ677909	Schoch et al. 2006
*** Stemphylium vesicarium ***	**CBS 191.86**	GU238160	GU238232	EF452449	DQ471090	Spatafora et al. 2006, Andrie et al. 2008, Aveskamp et al. 2010
*** Stemphylium botryosum ***	**CBS 714.68**	KC584345	KC584603	EF452450	DQ677888	Schoch et al. 2006, Andrie et al. 2008, Woudenberg et al. 2013
* Poaceicola arundinis *	MFLUCC 14-1060	KX655548	KX655553	KX655558	-	Hyde et al. 2016
* Poaceicola arundinis *	MFLU 16-0158	MG829057	MG829162	MG828947	MG829229	Wanasinghe et al. 2018
*** Poaceicola forlicesenica ***	**MFLUCC 15-0470**	KX910095	KX950406	KX926422	MG520922	Phookamsak et al. 2017, Thambugala et al. 2017
*** Poaceicola garethjonesii ***	**MFLUCC 15-0469**	KX954390	KY205717	KX926425	MG520923	Phookamsak et al. 2017, Thambugala et al. 2017
*** Populocrescentia ammophilae ***	**MFLUCC 17-0665**	MG829059	MG829164	MG828949	MG829231	Wanasinghe et al. 2018
*** Populocrescentia forlicesenensis ***	**MFLUCC 14-0651**	KT306952	KT306955	KT306948	MG520925	Ariyawansa et al. 2015a, Phookamsak et al. 2017
*** Populocrescentia rosae ***	**TASM 6125**	MG829060	MG829165	-	MG829232	Wanasinghe et al. 2018
*** Pseudoophiobolus achilleae ***	**MFLU 17-0925**	MG520966	-	MG520946	-	Phookamsak et al. 2017
*** Pseudoophiobolus galii ***	**MFLUCC 17-2257**	MG520967	MG520989	MG520947	MG520926	Phookamsak et al. 2017
*** Pseudoophiobolus urticicola ***	**KUMCC 17-0168**	MG520975	MG520996	MG520955	MG520933	Phookamsak et al. 2017
*** Pseudophaeosphaeria rubi ***	**MFLUCC 14-0259**	KX765299	KX765300	KX765298	-	Hyde et al. 2016
*** Pyrenochaeta nobilis ***	**CBS 407.76**	DQ678096	-	EU930011	DQ677936	Ferrer et al. 2006, Schoch et al. 2006
* Pyrenophora bromi *	DAOM 127414	JN940074	JN940954	JN943666	-	Schoch et al. 2012
* Pyrenophora dactylidis *	DAOM 92161	JN940087	-	JN943667	-	Schoch et al. 2012
*** Sclerostagonospora lathyri ***	**MFLUCC 14-0958**	MG829066	MG829170	MG828955	MG829235	Wanasinghe et al. 2018
*Sclerostagonospora* sp.	CBS 118152	JX517292	-	JX517283	-	Crous et al. 2012.
*** Scolicosporium minkeviciusii ***	**MFLUCC 12-0089**	KF366382	KF366383	-	-	Wijayawardene et al. 2013
*** Septoriella phragmitis ***	**CPC 24118**	KR873279	-	KR873251	-	Crous et al. 2015c
* Setomelanomma holmii *	CBS 110217	GQ387633	GQ387572	KT389542	GU349028	Schoch et al. 2009, de Gruyter et al. 2010, Chen et al. 2015
*** Setophoma chromolaena ***	**CBS 135105**	KF251747	-	KF251244	-	Quaedvlieg et al. 2013
*** Setophoma sacchari ***	**CBS 333.39**	GQ387586	GQ387525	KF251245	-	de Gruyter et al. 2010
* Setophoma sacchari *	MFLUCC 12-0241	KJ476147	KJ476149	KJ476145	KJ461318	Phookamsak et al. 2014b
* Setophoma sacchari *	MFLUCC 11-0154	KJ476146	KJ476148	KJ476144	KJ461319	Phookamsak et al. 2014b
*** Setophoma vernoniae ***	**CPC 23123**	KJ869198	-	KJ869141	-	Crous et al. 2014
* Staurosphaeria rhamnicola *	MFLUCC 17-0813	MF434288	MF434376	MF434200	MF434462	Wanasinghe et al. 2017
*** Staurosphaeria rhamnicola ***	**MFLUCC 17-0814**	MF434289	MF434377	MF434201	MF434463	Wanasinghe et al. 2017
* Sulcispora pleurospora *	CBS 460.84	-	-	AF439498	-	Câmara et al. 2002
*** Sulcispora supratumida ***	**MFLUCC 14-0995**	KP271444	KP271445	KP271443	-	Senanayake et al. 2018
*** Tintelnotia destructans ***	**CBS 127737**	KY090664	KY090698	KY090652	-	Ahmed et al. 2016
*** Tintelnotia opuntiae ***	**CBS 376.91**	GU238123	GU238226	KY090651	-	Aveskamp et al. 2010, Ahmed et al. 2016
*** Vagicola chlamydospora ***	**MFLUCC 15-0177**	KU163654	KU163655	KU163658	-	Jayasiri et al. 2015
*** Vrystaatia aloeicola ***	**CBS 135107**	KF251781	-	KF251278	-	Quaedvlieg et al. 2013
*** Wojnowicia italica ***	**MFLUCC 13-0447**	KX430001	KX430002	KX342923	KX430003	Hyde et al. 2016
*** Wojnowicia lonicerae ***	**MFLUCC 13-0737**	KP684151	KP684152	KP744471	-	Liu et al. 2015
*** Wojnowiciella dactylidis ***	**MFLUCC 13-0735**	KP684149	KP684150	KP744470	-	Liu et al. 2015
*** Wojnowiciella eucalypti ***	**CPC 25024**	KR476774	-	KR476741	-	Crous et al. 2015a
*** Wojnowiciella spartii ***	**MFLUCC 13-0402**	KU058729	MG520998	KU058719	MG520937	Li et al. 2015, Phookamsak et al. 2017
* Xenoseptoria neosaccardoi *	CBS 120.43	KF251783	-	KF251280	-	Quaedvlieg et al. 2013
*** Xenoseptoria neosaccardoi ***	**CBS 128665**	KF251784	-	KF251281	-	Quaedvlieg et al. 2013
* Yunnanensis phragmitis *	MFLUCC 17-0315	MF684863	MF684867	MF684862	MF683624	Karunarathna et al. 2017
*** Yunnanensis phragmitis ***	**MFLUCC 17-1361**	MF684865	MF684864	MF684869	-	Karunarathna et al. 2017

Maximum likelihood analysis was generated by using the CIPRES Science Gateway web server (Miller et al. 2010) and chosen RAxML-HPC BlackBox (8.2.10) (Stamatakis 2014). Maximum parsimony analysis was performed by PAUP v. 4.0b10 (Swofford 2002) with the heuristic search option with 1,000 random sequence additions and tree-bisection reconnection (TBR) as branch-swapping algorithm. All characters were unordered and of equal weight and gaps were regarded as missing data. Maxtrees were set up to 1,000, a zero of maximum branches length was collapsed and all multiple parsimonious trees were saved. Tree length [TL], consistency index [CI], retention index [RI], relative consistency index [RC] and homoplasy index [HI] were determined under different optimality criteria. The robustness was assessed using bootstrap analysis with 1,000 replications (Hillis and Bull 1993). The Kishino-Hasegawa tests were made in order to determine whether trees were significantly different (Kishino and Hasegawa 1989).

Bayesian inference analysis was conducted with MrBayes v. 3.2.2 (Ronquist et al. 2012) and a Bayesian posterior probability (BYPP) was determined by Markov Chain Monte Carlo sampling (MCMC). The Bayesian parameters were set up to “Lset applyto= (all) nst=6 rates=invgamma; prset applyto= (all) statefreqpr=dirichlet (1,1,1,1)”. Six simultaneous Markov chains were set up to 10,000,000 generations and trees were sampled every 100^th^ generation. The programme was automatically terminated when the average standard deviation of split frequencies reached below 0.01 (Maharachchikumbura et al. 2015). The distribution of log-likelihood scores were examined to determine the stationary phase for each search and to decide if extra runs were required to achieve convergence, using Tracer v.1.6 program (Rambaut et al. 2013). The first 10% of generated trees representing the burn-in phase were discarded and the remaining trees were used to calculate posterior probabilities of the majority rule consensus tree.

The tree was made in FigTree v. 1.4.3 (Rambaut 2016) and edited in Adobe Illustrator CS6 (Adobe Systems Inc., United States). The finalised alignment and tree were submitted in TreeBASE, submission ID: 23697 (http://www.treebase.org).

**Notes.** Ex-type strains are given in bold and the new species in this study is in red. “-” means that the sequence is missing or unavailable.

****Abbreviations.**AFTOL**: Assembling the Fungal Tree of Life; **CBS**: Westerdijk Fungal Biodiversity Institute, Utrecht, Netherlands; **CCTU**: Culture Collection of Tabriz University, Tabriz, Iran; **CPC**: Culture Collection of P.W. Crous; **DAOM**: Plant Research Institute, Department of Agriculture (Mycology), Ottawa, Canada; **JK**: J. Kohlmeyer; **KUMCC**: Kunming Institute of Botany Culture Collection, Chinese Academy of Sciences, Kunming, China; **MFLU**: Herbarium of Mae Fah Luang University, Chiang Rai, Thailand; **MFLUCC**: Mae Fah Luang University Culture Collection, Chiang Rai, Thailand; **TASM**: Tashkent Mycological Herbarium, Institute of Botany and Zoology, Uzbek Academy of Science, Uzbekistan; **UTHSC**: Fungus Testing Laboratory of the University of Texas Health Science Center at San Antonio, San Antonio, Texas, USA.

## Results

### Phylogenetic analyses

**Figure 1.**
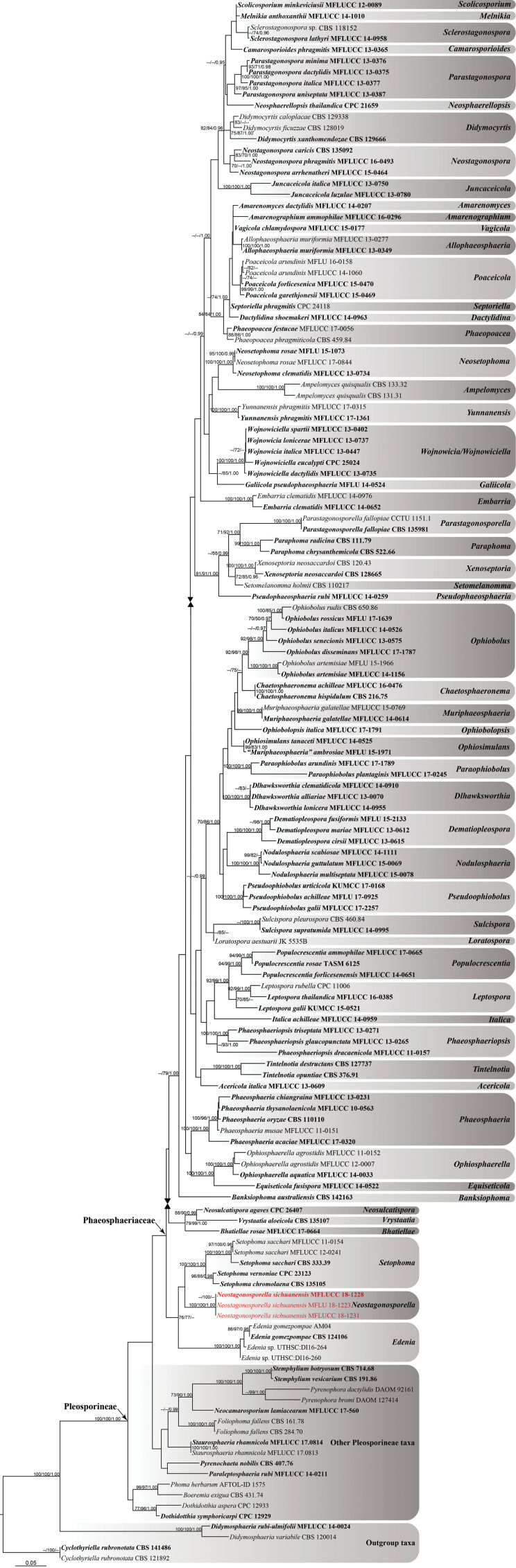
Phylogram generated from maximum likelihood analysis (RAxML) based on combined LSU, SSU, ITS and TEF 1-α sequenced data of taxa from the family Phaeosphaeriaceae and other representative species in Pleosporineae and Massarineae. The tree is rooted to *Cyclothyriellarubronotata* (CBS 121892), *C.rubronotata* (CBS 141486), *Didymosphaeriarubi-ulmifolii* (MFLUCC 14-0024) and *D.variabile* (CBS 120014). Bootstrap support values of maximum parsimony and maximum likelihood (MPBP, left; MLBP, middle) equal to or greater than 70% and Bayesian posterior probabilities (BYPP, right) equal to or greater than 0.95 are provided. The type strains were highlighted in bold and the newly generated sequences are highlighted in red.

In this phylogenetic analysis, we include all representative sequences of genera in Phaeosphaeriaceae and other representative genera and species in Pleosporineae and Massarineae. The final concatenated dataset containing 138 ingroup taxa within the suborder Pleosporineae, included 56 currently existing genera in Phaeosphaeriaceae, with 3559 characters including gaps (917 characters for LSU, 1046 for SSU, 681 for ITS and 915 for TEF 1-α). Single gene datasets of LSU, SSU, ITS and TEF 1-α were initially analysed and checked for topological congruence but these were not significantly different (data not shown). Support values of MP, ML and BI analyses (equal to or higher than 70% for MPBP and MLBP and 0.95 for BYPP) are shown in Fig. 1 which is the best scoring tree generated from ML. The phylogenetic trees generated from ML analyses were similar to previous phylogenies including Phaeosphaeriaceae (Phookamsak et al. 2014a, b, 2017, Jayasiri et al. 2015, Li et al. 2015, Liu et al. 2015, Phukhamsakda et al. 2015, Tibpromma et al. 2015, 2016, 2017, Hyde et al. 2016, Mapook et al. 2016, Ahmed et al. 2017, Huang et al. 2017, Karunarathna et al. 2017, Thambugala et al. 2017, Ariyawansa et al. 2018, Bakhshi et al. 2018, Senanayake et al. 2018, Wanasinghe et al. 2018).

The best scoring RAxML tree with the final optimisation had a likelihood value of -32702.569414. The matrix had 1387 distinct alignment patterns and 32.39% in this alignment is the gaps and completely undetermined characters. Estimated base frequencies were as follows: A=0.244424, C=0.233850, G=0.265929, T=0.255797, with substitution rates AC=1.171601, AG=2.805496, AT=2.145028, CG=0.771605, CT=6.035018 and GT=1.000000. The gamma distribution shape parameter α=0.167161 and the Tree-Length=5.334112. The maximum parsimony dataset consisted of 3559 characters, of which 2580 characters were constant, 217 were parsimony-uninformative and 762 were parsimony-informative. All characters were of type ‘unord’ with equal weight. The parsimony analysis resulted in a thousand equally most parsimonious trees with a length of 5829 steps (CI = 0.270, RI = 0.654, RC = 0.177, HI = 0.730). Bayesian posterior probabilities were determined by MCMC and the final average standard deviation of split frequencies was 0.009939.

*Neostagonosporellasichuanensis* clusters in the family Phaeosphaeriaceae with strong support (100% MLBP/100% MPBP/1.00 BYPP) and nucleotide sequences from all strains are the same and it confirms that our three collections are the same species. The multigene analyses show that *N.sichuanensis* is phylogenetically close to the genus *Setophoma* and *Edenia* and separated from the remaining genera of the family in a distinct clade with moderate bootstrap support.

### Taxonomy

#### Neostagonosporella

Taxon classificationFungiPleosporalesPhaeosphaeriaceae

C.L. Yang, X.L. Xu & K.D. Hyde gen. nov.

Index Fungorum number: IF555713

Facesoffungi number: FoF 05490

##### Type species.

*Neostagonosporellasichuanensis* C.L. Yang, X.L. Xu & K.D. Hyde

##### Etymology.

Name reflects the morphological similarity to the genus *Stagonospora*.

##### Description.

*Parasitic* on living to nearly dead stems and branches of bamboo. ***Sexual morph***: *Ascostromata* coriaceous, visible as raised to superficial on host, gregarious, multi-loculate, ellipsoidal, globose to subglobose or irregular in shape, dark brown to black, glabrous. *Locules* globose to subglobose, with a centrally located ostiole, lacking periphyses. *Peridium* multi-layered, of brown to dark brown, pseudoparenchymatous cells of *textura angularis*. *Hamathecium* comprising trabeculate, anastomosed pseudoparaphyses. *Asci* 8-spored, bitunicate, fissitunicate, cylindrical to cylindric-clavate, short pedicellate, apically rounded with an ocular chamber. *Ascospores* overlapping bi-seriate, hyaline, cylindrical to fusiform, septate, smooth-walled, surrounded by a distinct mucilaginous sheath. ***Asexual morph***: Coelomycetous. *Conidiostromata* pycindial, coriaceous, superficial, dark brown to black, fusiform to long fusiform or rhomboid, multi-loculate, solitary, glabrous. *Pycnidia* globose to subglobose, ostiolate. *Pycnidial wall* comprising multi-layered, of dark brown to black, pseudoparenchymatous cells of *textura angularis*. *Conidiophores* reduced to conidiogenous cells. *Conidiogenous cells* ampulliform to subcylindrical, smooth, hyaline, enteroblastic, phialidic, arising from inner layer of pycnidial wall. *Macroconidia* hyaline, subcylindrical to cylindrical, septate, nearly equidistant between septa, smooth-walled, sometimes surrounded by a mucilaginous sheath when immature. *Microconidia* hyaline, varied in shape, aseptate, smooth-walled, with small guttulate.

##### Notes.

*Stagonospora* resembles *Neostagonosporella* in asexual status, but *Stagonospora* differs in having generally uni-loculate conidiomata, a thick-walled pycnidial wall, doliiform, holoblastic conidiogenous cells with several percurrent proliferations at the apex and mostly smooth to verruculose conidia (Quaedvlieg et al. 2013, Hyde et al. 2016). Phylogenetic analyses based on a concatenated LSU, SSU, ITS and TEF 1-α sequence data (Fig. 1) show that *Neostagonosporella* is closely related to *Setophoma* and *Edenia* within Phaeosphaeriaceae. There are some significant differences in morphology between these genera and these are summarised in Table 2. Six species are currently accepted in *Setophoma* and two species in *Edenia* and both of them occur on different grasses but only our new collections are parasitic on bamboo. Comparison of DNA sequence data across four gene regions reveals base pair differences as shown in Table 3. Phylogenetic analyses also clearly differentiate these taxa (Fig. 1). It is the first time that species with massarineae-like morphology occurring on bamboo, were found in the Phaeosphaeriaceae. Based on molecular phylogeny, the new genus is introduced in Phaeosphaeriaceae to accommodate a massarineae-like taxon.

**Table 2.** Morphological comparison of *Neostagonosporella*, *Setophoma* and *Edenia*.

Morphology	* Neostagonosporella *	* Setophoma *	* Edenia *
	(Type: *N.sichuanensis*)	(Type: *S.terrestris*)	(Type: *E.gomezpompae*)
Ascostromata	Multi-loculate, globose to subglobose or irregular	Uni-loculate, globose	
Locules	Globose to subglobose, with a central ostiole, lacking periphyses	Globose, with a central ostiole	
Pseudoparaphyses	Narrow, septate, trabeculae, longer than asci	Broad, septate, prominently branched, constricted at septa, sometimes anastomosing	
Asci	Cylindrical to cylindric-clavate, short-pedicellate	Cylindrical or subcylindrical, fasciculate, pedicellate	
Ascospores	Bi-seriate, hyaline, cylindrical to fusiform, smooth-walled, transversely multi-septate	Uni- to multi-seriate, light brown or red brown, fusiform, sometimes verruculose, 2–3-septate	
Conidiostromata	Multi-loculate	Uni-loculate	
Pycnidia	Globose to subglobose, smooth, ostiolate	Globose to subglobose, setose, with papillate ostiolate	
Conidia	Two types. Macroconidia subcylindrical to cylindrical, transversely multi-septate, hyaline. Microconidia oval, ellipsoidal or long ellipsoidal, aseptate, hyaline	One type. Ellipsoidal to subcylindrical to subfusoid, aseptate, hyaline	One type. Ellipsoidal or slightly narrowed at base, aseptate, subhyaline
Others	On PDA, grey white, reverse dark brown. Hyphae developing by different angle branched and without forming rope-like strands	On PDA, iron-grey-olivaceous, reverse same. Hyphae undescribed	On PDA, pinkish-white, reverse reddish-brown, velvety to floccose. Hyphae frequently developing by 90° angle branched and forming rope-like strands
References	This study	de Gruyter et al. 2010, Quaedvlieg et al. 2013, Phookamsak et al. 2014a, b, Crous et al. 2016, Thambugala et al. 2017	González et al. 2007, Sun et al. 2013

**Table 3.** Comparison of DNA sequence data *Parastagonosporella* vs *Edenia* and *Setophoma*.

**Gene region**	***Parastagonosporella* vs *Edenia***	***Parastagonosporella* vs *Setophoma***
LSU	12/819 (1.47%)	13/818 (1.6%)
SSU	NA*	4/981 (0.4%)
TEF	47/869 (5.41%)	43/868 (5%)
ITS	89/515 (17.28%)	66/515 (12.8%)

*SSU is not available for *Edenia*

#### Neostagonosporella sichuanensis

Taxon classificationFungiPleosporalesPhaeosphaeriaceae

C.L. Yang, X.L. Xu & K.D. Hyde sp. nov.

Index Fungorum number: IF555714

Facesoffungi number: FoF 05491

Figs 2 , 3

##### Type.

CHINA, Sichuan Province, Ya’an City, Yucheng District, Kongping Township, Alt. 1133 m, 29°50.14'N 103°03'E, on living to nearly dead branches of *Phyllostachysheteroclada* Oliv. (Poaceae), 8 April 2016, C.L. Yang and X.L. Xu, YCL201604001 (MFLU 18-1212/SICAU 16-0001, **holotype**), ex-type living culture, MFLUCC 18-1228/SICAUCC 16-0001; Sichuan Province, Ya’an City, Yucheng District, Yanchang Township, Alt. 951 m, 29°43.57'N 103°04.74'E, on nearly dead stems of *Phyllostachysheteroclada* Oliv. (Poaceae), 9 April 2017, C.L. Yang and X.L. Xu, YCL201704001 (MFLU 18-1220/SICAU 17-0001, **paratype**), ex-type living culture, MFLUCC 18-1231/SICAUCC 17-0001; Sichuan Province, Ya’an City, Lushan County, Longmen Township, Alt. 949 m, 30°15.74'N 102°59.27'E, on nearly dead branches of *Phyllostachysheteroclada* Oliv. (Poaceae), 12 September 2017, C.L. Yang and X.L. Xu, YCL201709002 (MFLU 18-1223, **paratype**).

##### Etymology.

in reference to Sichuan Province where the specimens were collected.

##### Description.

*Associated* with stem spot disease on living to nearly dead stems and branches of *Phyllostachysheteroclada* (Poaceae). ***Sexual morph***: *Ascostromata* (0.5–) 1–2 (–4.5) × 0.8–1.3 mm long (*x*¯ = 1.9 × 1 mm, n = 50), 230–340 μm high (*x*¯ = 290 μm, n = 20), ellipsoidal, globose to subglobose or irregular in shape, immersed in host epidermis, becoming raised to superficial, coriaceous, solitary to gregarious, multi-loculate, erumpent through host tissue, with dark brown to black, glabrous, ostiole, usually generating subrhombic to rhombic pale yellow stripes at ascostromatal fringe. *Locules* 230–300 μm high (*x*¯ = 264 μm, n = 20), 330–460 μm diam. (*x*¯ = 393 μm, n = 20), clustered, gregarious, globose to subglobose, with a centrally located ostiole, lacking periphyses. *Peridium* 18–35 μm wide (*x*¯ = 27 μm, n = 20), composed of several layers of small, brown to dark brown pseudoparenchymatous cells of *textura angularis*, with inner hyaline layer, slightly thin at base, thick at sides towards apex, upper part fused with host tissue. *Hamathecium* composed of 1–2 μm (*x*¯ = 1.59 μm, n = 50) wide, filiform, septate, trabeculate, anastomosed pseudoparaphyses, embedded in a hyaline gelatinous matrix. *Asci* 90–125 × 12.5–14 μm (*x*¯ = 108.1 × 13.3 μm, n = 40), 8-spored, bitunicate, fissitunicate, cylindrical to cylindric-clavate, short pedicellate, 7.8–14 μm long (*x*¯ = 11 μm, n=20), apically rounded with an ocular chamber. *Ascospores* 30–35 × 6–7 μm (*x*¯ = 31.9 × 6.6 μm, n = 50), overlapping bi-seriate, hyaline, cylindrical to fusiform or subcylindric-clavate, with rounded to acute ends, narrower towards end cells, sometimes narrower at lower end cell, straight or slightly curved, 5–8 transversely septa, mostly 7-septate, slightly constricted at septa, nearly equidistant between septa, guttulate, smooth-walled, surrounded by a mucilaginous sheath, 5–9 μm thick (*x*¯ = 6.9 μm, n = 30). ***Asexual morph***: Coelomycetous. *Conidiostromata* 9–13 × 1–2 mm long (*x*¯ = 11.2 × 1.6 mm, n = 10), 320–350 μm high (*x*¯ = 332 μm, n=10), fusiform to long fusiform or rhomboid, coriaceous, superficial, dark brown to black, multi-loculate, solitary, scattered, glabrous. *Pycnidia* 180–240 μm high (*x*¯ = 209 μm, n = 20), 170–240 μm diam. (*x*¯ = 210 μm, n = 20), globose to subglobose, ostiolate. *Pycnidial wall* 12–18 (–23) μm wide (*x*¯ = 15 μm, n = 20), comprising multi-layered, brown to dark brown pseudoparenchymatous cells, of *textura angularis*, paler towards inner layers, slightly thin at base, thick at sides towards apex, upper part fused with host tissue. *Conidiophores* reduced to conidiogenous cells. *Conidiogenous cells* 3–5.5 (–7) × 3–4 μm (*x*¯ = 4.17 × 3.29 μm, n = 20), ampulliform to subcylindrical, smooth, hyaline, enteroblastic, phialidic, formed from inner layer of pycnidial wall. *Macroconidia* (32.5–) 33.5–40 (–44) × (5–) 5.5–7 (–7.5) μm (*x*¯ = 37.5 × 6.2 μm, n = 40), subcylindrical to cylindrical, narrowly rounded at both ends, sometimes curved, 7–13 transversely septa, nearly equidistant between septa, hyaline, smooth-walled, guttulate, sometimes surrounded by a mucilaginous sheath when immature. *Microconidia* (3–) 3.5–4 (–5) × (1–) 1.5–2 (–3) μm (*x*¯ = 3.9 × 1.9 μm, n = 50), oval, ellipsoidal or elongate-ellipsoidal, aseptate, rounded at both ends, hyaline, smooth-walled, with small guttulate.

**Figure 2.**
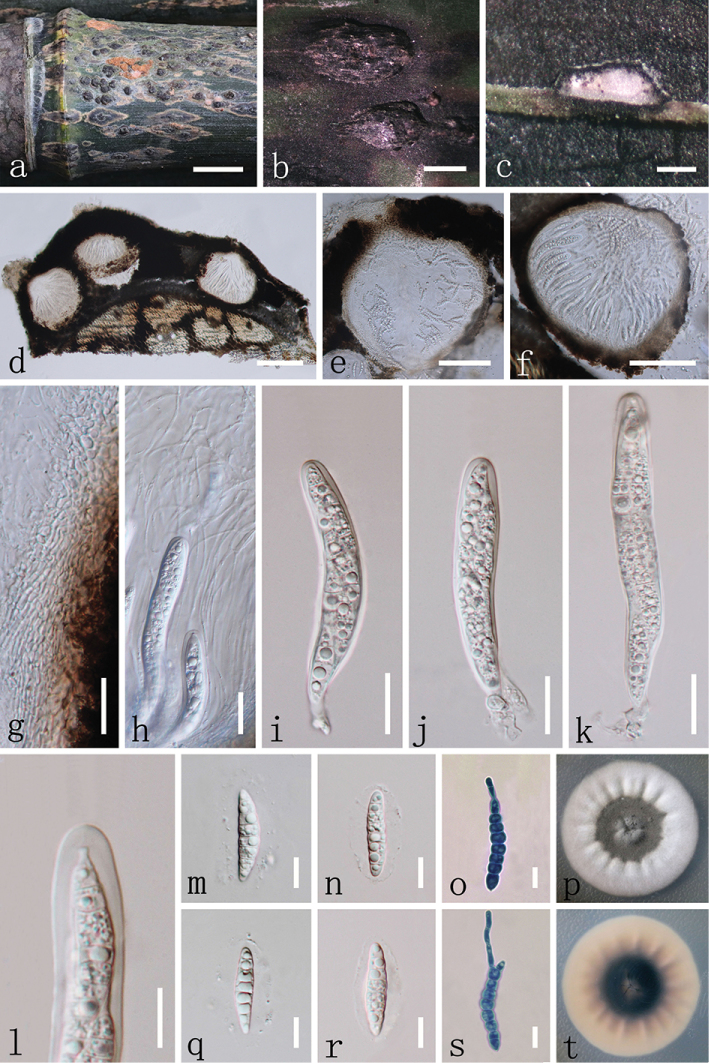
*Neostagonosporellasichuanensis* (MFLU 18-1212, holotype). **a** аppearance of ascostromata on host **b** ascostroma **c, d** vertical section of ascostroma **e, f** close up of ascoma **g** peridium **h** trabeculate pseudoparaphyses and asci **i–k** asci **l** bitunicate asci, note ocular chamber **m, n, q, r** ascospores with mucilaginous sheath **o, s** germinated ascospores in lactate cotton blue reagent **p, t** colonies on PDA (p-from above, t-from below). Scale bars: 1 cm (**a**); 1 mm (**b**); 200 μm (**c, d**); 100 μm (**e, f**); 20 μm (**g–k**); 10 μm (**l–o, q–s**).

**Figure 3.**
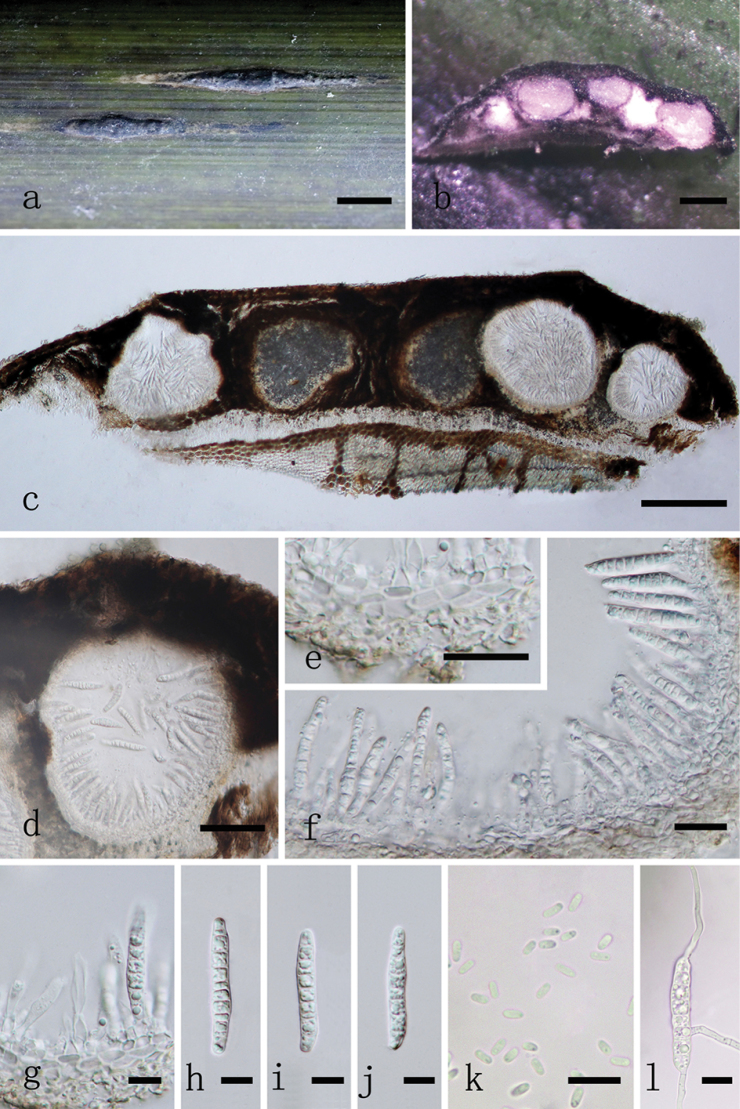
*Neostagonosporellasichuanensis* (MFLU 18-1220, paratype). **a** appearance of conidiomata on host **b, c** vertical section of conidioma **d** pycnidia **e** peridium **f, g** conidiogenous cells and developing conidia **h–l** conidia **m** germinated conidium. Scale bars: 1 cm (**a**); 200 μm (**b–d**); 20 μm (**e, f**); 10 μm (**g–m**).

**Culture characteristics.** Ascospores germinating in sterilised water within 24 hours at 25°C, with germ tubes developed from each cell of ascospores, mostly from middle and end of spores. Colonies on PDA circular, with concentric circles, grey white in outer side, fawn in reverse side, grey in inner side, dark brown on back side. Conidial germination similar to ascospores. Conidiomata formed on PDA at 25°C after 75 days, pycnidial, solitary to gregarious, raised on agar, black dots, pyriform, globose to subglobose, or irregular, uniloculate, covered by white or grey hyphae. Conidia two types, macroconidia and microconidia and both longer than ones on host. Macroconidia (30–)40–48(–60.5) × (4–)5–6 μm (*x*¯ = 43.8 × 5.2 μm, n = 50), hyaline, 4–7-septate, occasionally 3-septate, hyaline. Microconidia (3.5–)4–6(–12) × (1–)1.5–2(–3) μm (*x*¯ = 5.3 × 1.9 μm, n = 50), aseptate, hyaline.

## Discussion

*Neostagonosporella* has a unique suite of characters that differentiate it from other genera in Phaeosphaeriaceae, such as multi-loculate ascostromata and trabeculate pseudoparaphyses. Trabeculate pseudoparaphyses have been shown to be uninformative at the higher taxonomic levels (Liew et al. 2000), but appear to be informative at the genus level. *Neostagonosporella* is the only genus of Phaeosphaeriaceae with this type of pseudoparaphyses. Phaeosphaeriaceous taxa have diverse morphological characteristics and the familial placement of some genera could not be resolved based on a concatenated phylogeny of three to four loci, because some genera contain only 1-2 described species (Crous et al. 2015a, 2015b, 2017a, Jayasiri et al. 2015, Phukhamsakda et al. 2015, Tibpromma et al. 2015, 2017, Abd-Elsalam et al. 2016, Hernández-Restrepo et al. 2016, Hyde et al. 2016, 2017, Wijayawardene et al. 2016, Ahmed et al. 2017, Karunarathna et al. 2017, Phookamsak et al. 2017, Bakhshi et al. 2018, Wanasinghe et al. 2018).

Species of Phaeosphaeriaceae have been found on various hosts and substrates, including plants, lichens, mushrooms, algae, human, soil and air (Saccardo 1883, Berlese and Voglino 1886, Phookamsak et al. 2014a, Ahmed et al. 2016, Karunarathna et al. 2017, Zhang et al. 2017, Joshi et al. 2018). However, most Phaeosphaeriaceous genera occur on plants of more than 65 host families, the majority of them being monocotyledons and herbaceous plants, such as Arecaceae, Asparagaceae, Compositae, Juncaceae, Leguminosae, Poaceae, Ranunculaceae, Restionaceae and Rosaceae etc. (Taylor and Hyde 2003, Quaedvlieg et al. 2013, Crous et al. 2015b, Hyde et al. 2016, Tibpromma et al. 2016a, Karunarathna et al. 2017, Phookamsak et al. 2017, Wanasinghe et al. 2018). Our new genus exists on Poaceae and at least 30 genera are reported within this family. Currently, 11 genera are observed only on Poaceae: *Amarenomyces*, *Bricookea*, *Camarosporioides*, *Dactylidina*, *Embarria*, *Melnikia*, *Neosphaerellopsis*, *Phaeopoacea*, *Sulcispora*, *Vagicola* and *Yunnanensis*, all of them being recently established except for *Amarenomyces*, *Bricookea* and *Sulcispora* (Eriksson 1981, Barr 1982, Shoemaker and Babcock 1989, Trakunyingcharoen et al. 2014, Ariyawansa et al. 2015b, Hyde et al. 2016, Wijayawardene et al. 2016, Karunarathna et al. 2017, Thambugala et al. 2017, Wanasinghe et al. 2018). Amongst them, all hosts are short herbaceous plants and there are no bamboo plants recorded so far, with the exception of a few species of *Ophiobolus* and *Phaeosphaeria* in the old literature (Penzig and Saccardo 1897, Miyake and Hara 1910). A large number of bamboo forests (more than 130 species) are distributed throughout Sichuan (Yi 1997) and, most likely, many Phaeosphaeriaceae species are waiting for exploration and discovery.

## Supplementary Material

XML Treatment for Neostagonosporella

XML Treatment for Neostagonosporella sichuanensis
